# Anaphylactic reaction associated with Ranitidine in a patient with acute pancreatitis: a case report

**DOI:** 10.1186/1752-1947-1-75

**Published:** 2007-08-31

**Authors:** Ulfin Rethnam, Rajam Sheeja Yesupalan

**Affiliations:** 1 Department of Orthopaedics, Glan Clwyd Hospital, Rhyl, UK

## Abstract

Ranitidine is a widely used drug and is known to be well tolerated. This case report illustrates a severe anaphylactic reaction after a single intravenous dose of 50 mgs of ranitidine and highlights this unusual but life threatening adverse reaction.

**The patient:**

A 56 year old female with acute pancreatitis and a known allergy to metronidazole and buscopan developed an anaphylaxis few minutes following the injection of ranitidine for epigastric discomfort. She went on to develop anaphylactic shock and a cardiorespiratory arrest.

**Management:**

She was managed with adrenaline, hydrocortisone and ventilatory support following which she had a full recovery.

**Conclusion:**

Awareness of this rare but life threatening adverse reaction to a commonly used drug can avoid being caught unaware.

## Background

Ranitidine is a widely used H2 receptor antagonist and is usually well tolerated by patients. Being considered a safe drug, ranitidine is available over the counter all over the world. It is a drug commonly administered in the Accidents & Emergency. Anaphylactic reaction to ranitidine is rare. This life-threatening adverse reaction can take the clinician by surprise.

This case report highlights the occurrence of this rare but dangerous adverse reaction to ranitidine.

## Case presentation

A 56-year-old female with acute pancreatitis was admitted under our care. She was known to suffer from diverticular disease and had a myocardial infarction in the past. She was allergic to metronidazole and buscopan. She had no family history of drug allergies.

During the initial course of management she was given 50 mg of ranitidine as a slow intravenous bolus for epigastric discomfort. Few minutes after the injection, the patient complained of itching at the injection site that spread to involve the entire upper limb. She also complained of swelling of her tongue and difficulty in breathing. Within minutes her level of consciousness deteriorated and she became comatose. The initial examination revealed the following features: GCS 6/15, a grossly oedematous face, neck and extremities, a grossly swollen tongue, congested conjunctivae, cyanosis, diffuse rhonchi over both lung fields.

Despite immediate administration of intramuscular adrenaline, intravenous hydrocortisone and high flow oxygen the patient progressed into a cardio respiratory arrest. Cardiopulmonary resuscitation was commenced. Patient was intubated with difficulty and was resuscitated successfully. She was transferred to the Intensive Care Unit for ventilatory and inotropic support. Two days later she was weaned off the ventilator and by then she was haemodynamically stable without inotropic support. She made a full recovery in 3 days. A skin sensitivity test prior to the patient's discharge revealed the patient's sensitivity to Ranitidine.

## Discussion

Ranitidine is a H2 receptor antagonist widely used for acid peptic disease and usually well tolerated. Anaphylactic reaction to ranitidine is rare and only a few cases have been reported in the literature. Most of the patients reported were obstetric patients.[1-3] In our review of literature there have been no report of severe anaphylaxis to ranitidine in a patient with pancreatitis. Demirkan et al found only 2 cases of anaphylactic reaction due to ranitidine of 8304 first referral patients over a 13 year period. [4] The incidence of anaphylactic reaction to H2 receptor antagonists and proton pump inhibitors together has been reported as 0.3% – 0.7%. [5] We could not find any association between allergies to metronidazole and buscopan and the development of an anaphylactic reaction to ranitidine.

The anaphylactic reaction in our patient was due to ranitidine as she developed signs of anaphylaxis a few minutes after receiving the intravenous dose the drug. She was not given any other medication prior to ranitidine. The management was directed towards combating the severe anaphylactic reaction. All the cases reported in literature were treated along the same lines. No mechanisms have been identified for this adverse reaction. As the patient was known to be allergic to other medications, this may suggest that allergy to ranitidine may develop in patients with known multiple allergies.

This type of a reaction to a commonly used drug like ranitidine can take the clinician by surprise. Although the management for this is to treat the anaphylaxis, it is important that the clinician is aware of this adverse reaction.

## Conclusion

This case report was prepared to highlight a rare and unusual adverse reaction to a widely used drug, ranitidine. Caution needs to be exercised on intravenous administration of this drug as the physician can be caught unaware.

## Competing interests

The author(s) declare that they have no competing interests.

## Authors' contributions

UR was involved in collecting patient details, reviewing the literature and drafted the manuscript as the main author. RSY was involved in reviewing the literature and proof reading of the manuscript. RSY has approved the final manuscript. All authors have read and approved the final manuscript.
